# Cancer staging data collection using rules-based natural language processing for entity extraction from pathology notifications: the WA cancer staging project

**DOI:** 10.1186/s12911-026-03617-8

**Published:** 2026-06-15

**Authors:** Jade Newton, Jamiu Ekundayo, Nancy Tippaya, Shantelle Smith, Dagmawi Tadesse, Hamza Cakan, Rachael Moorin

**Affiliations:** 1 School of Population Health, Curtin University, Kent St, Bentley, WA 6102 Australia; 2 Faculty of Health Sciences, Curtin Health Innovation Research Institute, Curtin University, Bentley, WA 6102 Australia; 3 Curtin Institute for Data Science, Curtin University, Bentley, WA 6102 Australia; 4 WA Cancer Registry, Information and Performance Governance, Department of Health, East Perth, WA 6004 Australia; 5 School of Population and Global Health, The University of Western Australia, Crawley, WA 6009 Australia

**Keywords:** Neoplasm staging, Natural language processing, Breast neoplasms, Colorectal neoplasms, Melanoma

## Abstract

**Background:**

Cancer staging data is vital for treatment planning, outcome prediction, clinical research, and healthcare resource allocation. Collection at the population level can improve insights, however existing manual methods are resource intensive. This study aimed to develop rules-based natural language processing (NLP) systems to: (1) extract explicit tumour, node, and metastasis (TNM) entities from data reported to the Western Australian Cancer Registry; (2) extract implicit entities translatable to individual TNM values; and (3) translate these values into cancer stages for melanoma, breast, and colorectal cancers based on the AJCC 8th edition TNM staging system.

**Methods:**

Rules-based NLP systems were developed with extensive consultation from knowledge experts to extract staging information and stage colorectal, breast, and melanoma cancers using pathology reports and a hospital inpatient morbidity dataset. Their performance was evaluated against manual collections (ground truth) created by cancer staging project officers using recall, precision, and F1-scores.

**Results:**

After an iterative development process, the rules-based NLP systems correctly staged 87%-90% of cases compared to manual collections created by cancer staging officers. The melanoma NLP system had a weighted average precision of 0.96, recall of 0.94, and F1-score of 0.94. The colorectal and breast models had weighted average precision, recall, and F1-scores of 0.89, 0.89, 0.89; and 0.90, 0.89, and 0.89, respectively. The rules-based NLP systems demonstrated strong performance, with potential for improved accuracy using additional data sources.

**Conclusion:**

A rules-based NLP architecture can accurately derive TNM components and cancer stage from routinely collected clinical text. Whilst dependent on domain-expert input rather than data-driven training, the methods described demonstrate a way to support partial automation of cancer staging workflows in a setting with limited training datasets.

**Supplementary Information:**

The online version contains supplementary material available at 10.1186/s12911-026-03617-8.

## Background

Cancer staging information, which describes the size and spread of a cancer tumour, provides key information for treatment planning and prognosis [1]. At the population level, staging information provides insights into survival and treatment patterns and can inform service planning and policymaking decisions to improve the health outcomes of cancer patients across a population [2, 3]. The most commonly used staging system is the American Joint Commission on Cancer’s (AJCC) Tumour, Node, and Metastasis (TNM) classification system for solid tumours, applicable to most cancer types [4]. The TNM system describes the extent of cancer based on three criteria: **T (Tumour)**, which indicates the size, extent, and/or depth of the primary tumour (T), typically ranging from T1 to T4, with higher numbers reflecting larger tumour size or more extensive local invasion; **N (Nodes)**, which denotes the involvement of regional lymph nodes (N), ranging from N0 to N3, with higher numbers representing greater lymph node involvement; and **M (Metastasis)**, which signifies the presence of distant metastasis, categorised as M0 for no distant metastasis and M1 for the presence of distant metastasis. Further subclassifications of TNM and their definitions vary by cancer type, providing a more precise description of the extent and impact of cancer [4].

Cancer staging is generally documented by medical professionals as TNM, which combines individual TNM values. For example, a locoregional cancer that has metastasised to the lymph nodes but not to other parts of the body might be classified as T2N1M0, while a more advanced cancer that has spread to distant sites could be classified as T4N3M1. In this paper, we will refer to this comprehensive description of TNM as the ‘AJCC-TNM stage’. This AJCC-TNM is used to derive an overall stage group ranging from 0 to 4 (or 0 to IV). This summary level description is referred to as the ‘overall stage group’ throughout. Stage 0 indicates abnormal cells are present but have not breached the basement membrane and are in situ; Stage 1 indicates that cancer is present but has not spread to regional lymph nodes; Stages 2 and 3 indicate that the cancer is more invasive, larger, and/or has spread to regional lymph nodes or adjacent structures; and Stage 4 indicates that the cancer has metastasised to other parts of the body [4].

Pathology reports are produced by pathologists and provide detailed pathological information about cancer specimens obtained through various methods, including biopsies, fine needle aspirations, cytology specimens, and surgical resections. These reports often include data relevant to AJCC-TNM staging information, along with other variables essential for determining prognosis, progression and overall stage of cancer. In Australia, The Royal College of Pathologists of Australasia (RCPA) recommends the explicit reporting of AJCC-TNM staging in standardised pathology reports [5]. While individual TNM values and the overall stage group are sometimes reported explicitly according to RCPA protocols, reports are often unstructured and the way in which protocols have been implemented differ across labs. As a result, AJCC-TNM staging often needs to be inferred from textual descriptions of AJCC-TNM, such as tumour size or depth of invasion, the number of involved nodes, and evidence of metastatic spread, where available [6]. Due to their often unstructured nature, similar to other clinical reports, pathology reports are well-suited for natural language processing and/or machine learning (ML) data extraction techniques [7].

Automated data extraction from clinical free-text reports, often referred to as clinical NLP, has gained traction with researchers and clinicians seeking timely and reliable data to support quicker clinical decisions and policymaking relating to patients’ diagnoses and treatments [7]. Most published clinical NLP systems are rules-based [8]. Rules-based NLP systems are commonly used for labelling or annotating medical, radiology or pathology reports for applications such as developing decision support systems [9]. Traditionally, these systems require expert knowledge to be translated into word or character patterns or a list of search keywords (known as trigger phrases) [10], which are mined from unstructured medical or pathology reports [11]. Rules-based models are intuitive and explainable [12], and can achieve accuracies around 90%, with potential for improvement through further refinement of search keywords [13, 14]. However, the time saved on code development may be offset by the significant time required to create the search patterns. Additionally, these rules are often task-specific, limiting their generalisability across multiple tasks [11, 15, 16]. Thus, sustainability may become a concern when dealing with data from multiple cancer types. Automatic generation of trigger phrases or rules [17, 18] has been proposed as a potential solution to this issue, although the applicability of such automatic rule-generation to real cases remains uncertain [12].

Studies utilising ML and deep neural networks (DNN) have emerged as alternatives in recent years [19, 20]. These advanced models have proven useful in addressing some of the limitations of rules-based models [21]. They are task-agnostic, making them relatively more generalisable [22]. These models can be trained to extract entities related to cancer staging and determine the overall stage group without the need for explicit search patterns or rules [23]. However, reported F1-scores (model performance) for cancer-related studies with these advanced models range from 65% to 100% [23–26], indicating that they may not necessarily outperform rules-based models. Moreover, ML and DNN models require accurately labelled datasets for training, while rules-based NLP models have demonstrated reliability in generating such labelled datasets through information or entity extraction from unstructured medical reports [25, 27]. Given the absence of staged cancer cases to be used as training data, a rules-based NLP system was considered the best starting point for generating labelled data in consultation with domain experts to generate staged cancer data. The labelled data generated from rules-based NLP systems will be used for future model developments that may involve ML techniques.

In this study, rules-based NLP architecture was developed to extract specific entities from the pathology reports. Where explicit individual TNM values are not available in the reports, the rules-based systems extracted other relevant entities from textual descriptions and inferred the TNM values implicitly, following AJCC-TNM 8th edition rules specific to each cancer type. The resultant TNM values were then used to derive the overall stage group for each cancer. The TNM values and corresponding stages obtained from the rules-based systems were compared with a dataset of expert-labelled records created by cancer staging officers over one year for each cancer type to assess performance.

## Methods

This project was undertaken as part of the WA Cancer Staging Project, which was a collaboration between Curtin University and the Department of Health WA to develop methods to collect cancer staging data in Western Australia. It is noted that due to Intellectual Property constraints in the development of this body of work, the models resulting from the rules-based NLP architecture are not presented – instead the methods to develop them and their performance are described in detail.

### Data sources

Data was sourced from several routinely collected, WA Department of Health-managed data collections. These include the WA Cancer Registry (WACR) (Cancer Information System), and the Hospital Morbidity Data Collection (HMDC). Clinical coders within the Clinical Classifications Team at the Department of Health manually assign codes to cases in CanIS. The HMDC includes data coded at site/hospital by clinical coders, and monitored for quality and compliance by Senior Clinical Classification Officers within the Clinical Classifications Team. The data collections are described in more detail below.

#### The WA cancer registry

The WACR uses an internal software application, Cancer Information System (CanIS), that allows clinical coders at the Department to access pathology reports (notifications) of cancer patients in WA for reporting on population incidence, survival and mortality. Pathology providers are legislatively required to report malignant cancer notifications to the WACR [6]. These reports are imported and stored within CanIS and contain staging information. Since 2018, the WACR has manually collected staging information from explicit TNM statements in pathology reports during routine coding. However, this ceased with the start of the WA Cancer Staging Project, as the approach was not able to account for implicit TNM stage or correlations to assess the extent of disease.

For incidence collection, the clinical coders gather information on patient demographics, tumour-specific details, and diagnosis information, adhering to the International Classification of Diseases for Oncology (ICD-O) for coding cancer data. ICD-O codes provide a comprehensive characterisation of cancer tumours, including their topography (anatomical location), morphology and behaviour [28]. To ensure completeness where data is missing, the clinical coders may also review medical documentation from other systems if available, such as additional pathology reports, radiology reports, discharge summaries, and clinical correspondence, to support the ICD-O codes they record. The ICD-O codes in scope for each tumour stream are specified in the business rules (Supplementary material A).

#### Hospital morbidity data collection

The Hospital Morbidity Data Collection (HMDC) contains clinically coded data for inpatient episodes in public and private hospitals in WA. clinical coders at a hospital-level code inpatient episodes using the International Statistical Classification of Diseases and Related Health Problems, Tenth Revision, Australian Modification (ICD-10-AM). ICD-10-AM codes from the HMDC were used as supplementary information when information about the nodal involvements and/or metastasis were unavailable in pathology reports.

### Data collection

Data extraction followed the guidance of tumour-specific multidisciplinary project working groups (PWGs) formed to develop staging business rules in alignment with national and international best practice for the WACR [6]. The AJCC-TNM staging rules [29] and rules developed for a previous national staging project [30] were initially reviewed by project officers, and preliminary assumptions were made about how these rules could be applied to the data to extract staging information from CanIS and HMDC. These assumptions were then extensively discussed with the PWGs to come to agreement on whether these assumptions were appropriate for the available data sources. Once the staging business rules were finalised for each cancer type, they were used to inform the development of the code base for each tumour’s rules-based NLP system. The business rules are detailed in Supplementary material A.

The rules-based NLP system was deployed on registered cancers extracted from CanIS, where the ICD-O code indicated a primary diagnosis of breast, colorectal or melanoma cancer. Additionally, lymph node and distant metastasis reported within four months of the diagnosis date during the study period (1st January 2019 to 31st December 2019) were extracted from HMDC if the episode included an ICD-10-AM code for lymph node or distant metastasis.

#### Corpora

Manual staging cancer data from the pathology reports were collected for breast, colorectal, and melanoma cancers (the same input data source as the rules-based NLP system). The number of manually staged 2019 cases was as follows: melanoma (*N* = 1436), breast cancer (*N* = 1577), and colorectal cancer (*N* = 947). This manual staging process was conducted by cancer staging project officers, following rigorously defined, evidence-based business rules supported by the PWGs (Supplementary material A). A similar approach was used by Nguyen et al. [16] for their development.

### NLP system architecture

**Fig. 1:**
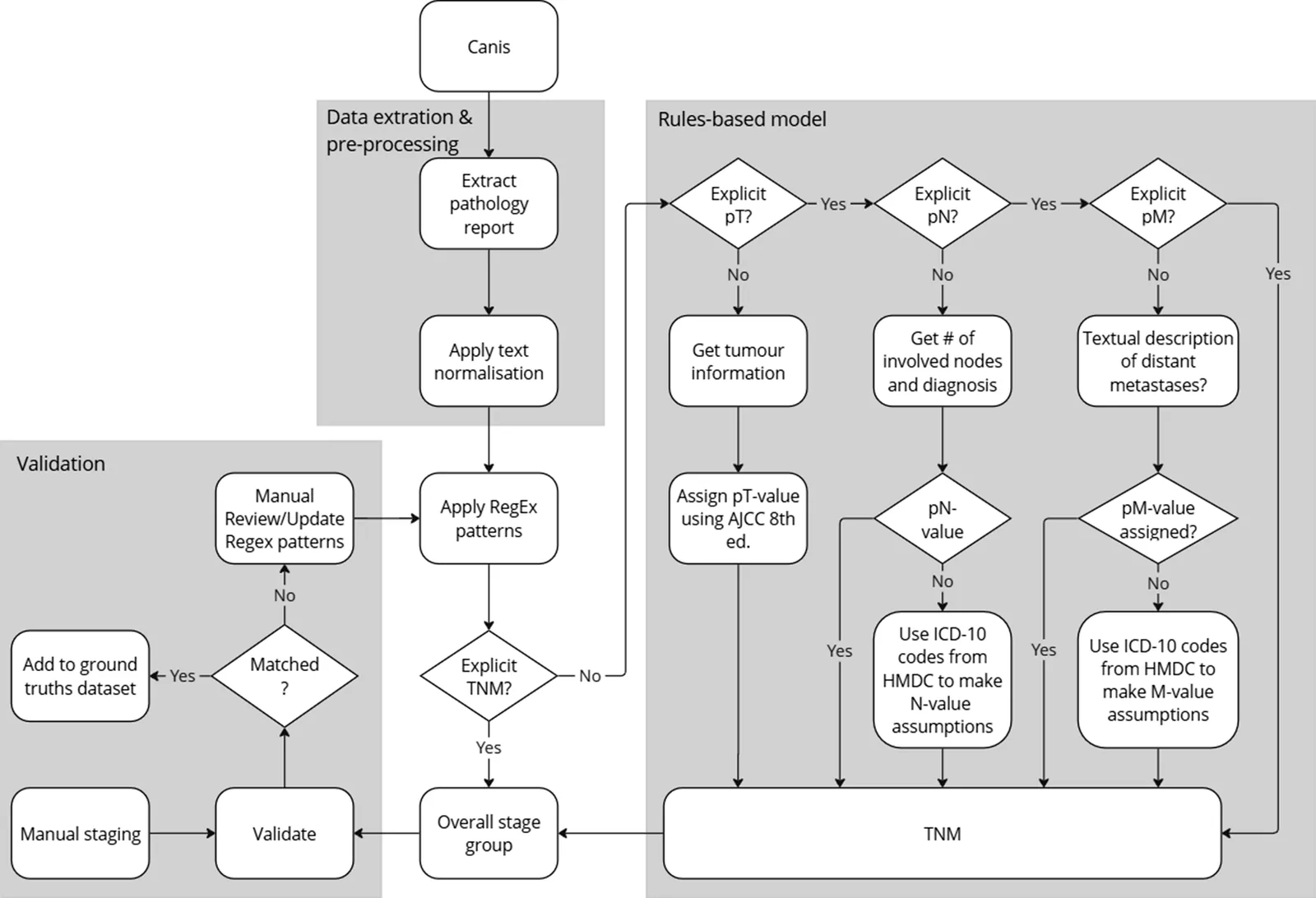
Cancer entity extraction and staging workflow

NLP system architecture was developed in Python. Figure 1 summarises the rules-based pipeline for extracting and assigning TNM values from pathology reports. The related pseudocode is available in Supplementary Material B. Figure 1 illustrates the data extraction initially undertaken from CanIS to obtain pathology reports, which is followed by text normalisation. Regular expression patterns are then applied to identify explicit TNM values. If no explicit staging information is identified, a deterministic system will infer T, N and M components using relevant information reported on the tumour, nodes, and metastasis, if available. These decisions are informed by the business rules that were developed in consultation with clinicians (Supplementary material A). Once TNM information has been extracted, an overall group stage is assigned. The final staging extracted by the system is then validated against the manually collected ground truth data, to iteratively refine regex patterns based on mismatches. This approach enables scalable, interpretable staging while maintaining traceability for validation and quality improvement. The process is described in more detail below.

#### Text pre-processing

Due to the unavailability of synoptic reporting and more importantly, to ensure minimum information loss, the workflow described above was implemented on each report as a single text. Initial data processing included converting characters to lower cases before applying a text normalisation. Text normalisation helped to remove any inconsistent texts of the same information entered by different pathologists, and ultimately improved the performance of the rules-based NLP system [10]. Some examples of numerous descriptions of “Breslow Thickness” found in melanoma reports are shown in Table 1.

**Table 1:** Example of normalised texts for melanoma

Original description	Normalised description
breslow microscopic thickness	breslow thickness
breslow measurement (thickness)	
thickness (breslow measurement)	
breslow tumour thickness	
dermal invasion with a thickness	
melanoma with a thickness	
melanocytes with a thickness	
melanoma cells a thickness	
melanocytic cells a thickness	
melanoma, the largest measuring	
melanoma, the largest of which is	
melanoma measuring	
melanoma. this extends to a depth	
melanoma with a depth	
dermal thickness	
dermis thickness	
tumour thickness	
breslow depth	

#### Rules-based NLP system & validation

As shown in Fig. 1, the system was designed to first extract any explicitly stated TNM values from each pathology report based on pre-defined search rules codified into regular expression patterns. If a TNM individual value is found, the complete AJCC-TNM is extracted (AJCC-TNM stage) and the overall stage group would be derived according to the AJCC-TNM rules.

Expert review by the cancer project staging officers of melanoma, breast, and colorectal cases demonstrated that explicit TNM values were not consistently referenceable in the corpus. Therefore, it was pertinent to use various descriptions and/or variables in the report to identify the *individual TNM values* implicitly. These descriptions were developed through extensive consultation with the cancer staging project officers, and tumour-specific PWGs where applicable. For example, in the case of melanoma, several parameters were identified that could be abstracted from to infer TNM values. These included the Breslow thickness (or tumour size) and ulceration level for melanoma tumours to infer the *T-values (*Table 1*)*; the number of positive or involved lymph nodes, diagnosis procedure, and the presence of satellite metastases or microsatellites to infer the *N-values*; and textual evidence of distant metastasis to determine the *M-value as M1* or *M0*. Lastly, *Tx*,* Nx* and/or *Mx* were used to denote the *T-value*,* N-value*, and *M-value* for cases where the desired entities were not found or the result of the extraction was inconclusive. For such cases, the presence of HMDC data with relevant ICD-10-AM codes, as determined through discussion with the tumour-specific PWGs, were used to represent lymph node invasion and infer the *N-value*. Similarly, the presence of HMDC data with relevant ICD-10-AM codes were used to infer *M-value*. The business rules developed for each tumour stream are available in Supplementary Material A. These business rules represent the final version of an iterative process refining rules to minimise missing values - the development process was iterative and exploratory, and detailed logs of each update were not retained.

#### Determination of overall stage group

Once the TNM values were determined, the AJCC-TNM rules specific to the cancer type (melanoma, breast, or colorectal cancer) were applied to derive the cancer stage (overall stage group). Table 2 summarises how the AJCC-TNM stage and overall stage groups were determined by combining the resultant values of TNM using the AJCC 8th edition classification system [29]. Note that for staging purposes, it was assumed that Tx = T0, Nx = N0 and Mx = M0. This assumption was necessary to increase the number of cases that can be staged [31]. This assumption has been adopted by other population-based cancer registries to deal with missing data and was made in consultation with PWGs [6].

**Table 2:** Stage determination from TNM values (Tx = T0, Nx = N0, Mx = M0)

Cancer Type	T	*N*	M	AJCC TNM stage	Overall stage group
Breast	T1*	N1	M0	Stage 1 A	Stage 1
	T0, T1	N1Mi		Stage 1B	
		N1		Stage 2 A	Stage 2
	T2	N0			
		N1		Stage 2B	
	T3	N0			
	T0, T1, T2	N2		Stage 3 A	Stage 3
	T3	N1			
	T4	N0, N1, N2		Stage 3B	
	Any T	Any N	M1	Stage 4	Stage 4
Colorectal	T1, T2	N0	M0	Stage 1	Stage 1
	T3			Stage 2 A	Stage 2
	T4a			Stage 2B	
	T4b			Stage 2 C	
	T1, T2	N1, N1a, N1b, N1c		Stage 3 A	Stage 3
	T1	N2, N2a, N2b		Stage 3	
	T2, T3	N2, N2a		Stage 3B	
	T3, T4, T4a	N1, N1a, N1b, N1c			
	T1, T2	N2b			
	T4a, T4	N2, N2a		Stage 3 C	
	T3, T4, T4a	N2b			
	T4b	N1, N1a, N1b, N1c, N2, N2a, N2b			
	Any T	Any N	M1	Stage 4	Stage 4
			M1a	Stage 4 A	
			M1b	Stage 4B	
			M1c	Stage 4 C	
Melanoma	T1, T2	N0	M0	Stage 1	Stage 1
	T1a, T1b			Stage 1 A	
	T2a			Stage 1B	
	T3, T4			Stage 2	Stage 2
	T2b, T3a			Stage 2 A	
	T3b, T4a			Stage 2B	
	T4b			Stage 2 C	
	T1, T1a, T1b, T2, T2a	N1a, N2a		Stage 3 A	Stage 3
	T0	N1b, N1c, N2b, N2c, N3b, N3c		Stage 3B	
	T1, T1a, T1b, T2, T2a	N1b, N1c, N2b			
	T2b, T3, T3a	N1a, N1b, N1c, N2a, N2b			
	T1, T1a, T1b, T2, T2a, T2b, T3, T3a	N2c, N3a, N3b, N3c		Stage 3 C	
	T3b, T4, T4a	Any N > N0			
	T4b	N1a, N1b, N1c, N2a, N2b, N2c, N3a, N3b, N3c			
	Any T	Any N	M1	Stage 4	Stage 4

Note. T1* for breast comprises T1Mi, T1a, T1b and T1c; for all cancers, T0N0M0/TisN0M0 = Unstageable = Stage 9

#### Validation

As a way of validation, the cancer stages determined from the workflow above were compared with the ground truth data for each cancer type to determine the overall performance of the developed NLP systems, and more importantly identify over-staged or under-staged cases. These cases were manually reviewed to improve performance. Reasons for misclassification were tumour-specific. For example, initially, the rules-based NLP system extracted TNM staging information for breast tumours that had undergone neoadjuvant treatment. As we are collecting stage at diagnosis, the ground truth data would have ignored this information and either reported any TNM information from the earliest pathology report, or reported it as missing if no TNM information was reported prior to treatment. Review of these cases allowed the project team to refine the regex pattern to identify and try to exclude information reported after neoadjuvant treatment.

This validation approach is continuous and adaptive, with the intention being that the proportion of cases staged is monitored. Dips in performance (proportion of cases staged) would trigger a case review by project officers to identify and improve the rules-based NLP system in response to any systematic changes in reporting that may occur.

#### Performance metrics

The performance of the rules-based systems were assessed using a combination of visual and statistical metrics. The visual measures are the bar-plots and the confusion matrices. A bar-plot compares the absolute counts of each class with the gold standard (the corresponding value from the manual collections). Confusion matrices provide a two-dimensional visualisation of the multiclass confusion matrix. Unlike bar-plots, confusion matrices can discriminate classes that are correctly matched with the gold standard from those that are discordant. With the inclusion of the percentage correctly and incorrectly matched for each class, the confusion matrices generated in this study provided visibility into bias towards any class.

The statistical metrics used to evaluate performance were precision, recall, F1-score, and overall accuracy, all of which were assessed from the classification reports method in Scikit-Learn python library [32]. Precision, P is the ratio of predicted true positives (TP) to the total number of positive predictions (that is, the sum of TP and false positives, FP). It measures the proportion of the positive predictions that are truly positive. Recall (R), on the other hand, is the ratio of the TP to the sum of TP and false negatives, FN. It is a measure of the actual positives correctly identified by the rules-based NLP system. Given the contrasting nature of precision and recall, a weighted harmonic mean known as *f1-score* is often used as a more reliable measure of performance.

$$\eqalign{& P = \>{{TP} \over {TP + FP}};\,\,R = \>{{TP} \over {TP + FN}};\> \cr & F1 = \>2 \times \>{{P \times \>\>R} \over {P + \>R}} \cr} $$

Lastly, the overall accuracy (*acc*) was determined as the ratio of the product of the f1-score (*f1*) for a given class and its frequency (*f*) to the total frequency. That is:

$$\>Acc = \>{{\sum {\>_{i = 1}^n} {{\left( {f1 \times \>f} \right)}_i}} \over {\sum {\>_{i = 1}^n} {f_i}i\> = \>1,\>2,\> \ldots \>,\>n}}$$

Where (n = number of classes).

## Results

Table 3 summarises the overall accuracies of the rules-based systems for extracting TNM and AJCC-TNM stage and Overall Stage Groups. Despite the melanoma rules-based NLP system having the lowest performance for T-values (F1-score = 0.90, vs. F1-scores of 0.92 and 0.95 for breast and colorectal rules-based NLP systems), the melanoma rules-based NLP system had the highest performances for both AJCC-TNM stage (F1-score = 0.91, vs. F1-scores of 0.87 and 0.88 for breast and colorectal rules-based NLP systems) and Overall stage groups (F1-score = 0.94, vs. F1-scores of 0.89 for both the breast and colorectal rules-based NLP system). This is partly due to the high performances achieved for both N and M, but more importantly, because the AJCC-TNM manuals’ decision rules for determining the cancer stage from the TNM values (Table 2) are more explicit compared to breast and colorectal cancers. The higher accuracies achieved for breast and colorectal for T and N values are attributable to the availability of explicit TNM values in some of their reports. Melanoma reports rarely include TNM values explicitly, but they contain detailed information that could be used to infer the value of T implicitly. Hence for melanoma, most of the T-values were determined implicitly from extracted tumour size and ulceration levels. Thus, the observed performances showed that the rules-based NLP systems were robust in extracting the individual subclasses of T-values for the different cancer types.

**Table 3:** Summary of rules-based system performances on 2019 datasets across all three cancer types

Cancer Type	Number of records validated against	T-values	*N*-values	M-values	AJCC TNM stage	Overall stage group
Breast	1577	0.92	0.95	0.97	0.87	0.89
Colorectal	947	0.95	0.97	0.89	0.88	0.89
Melanoma	1436	0.90	0.97	0.99	0.91	0.94

**Fig. 2:**
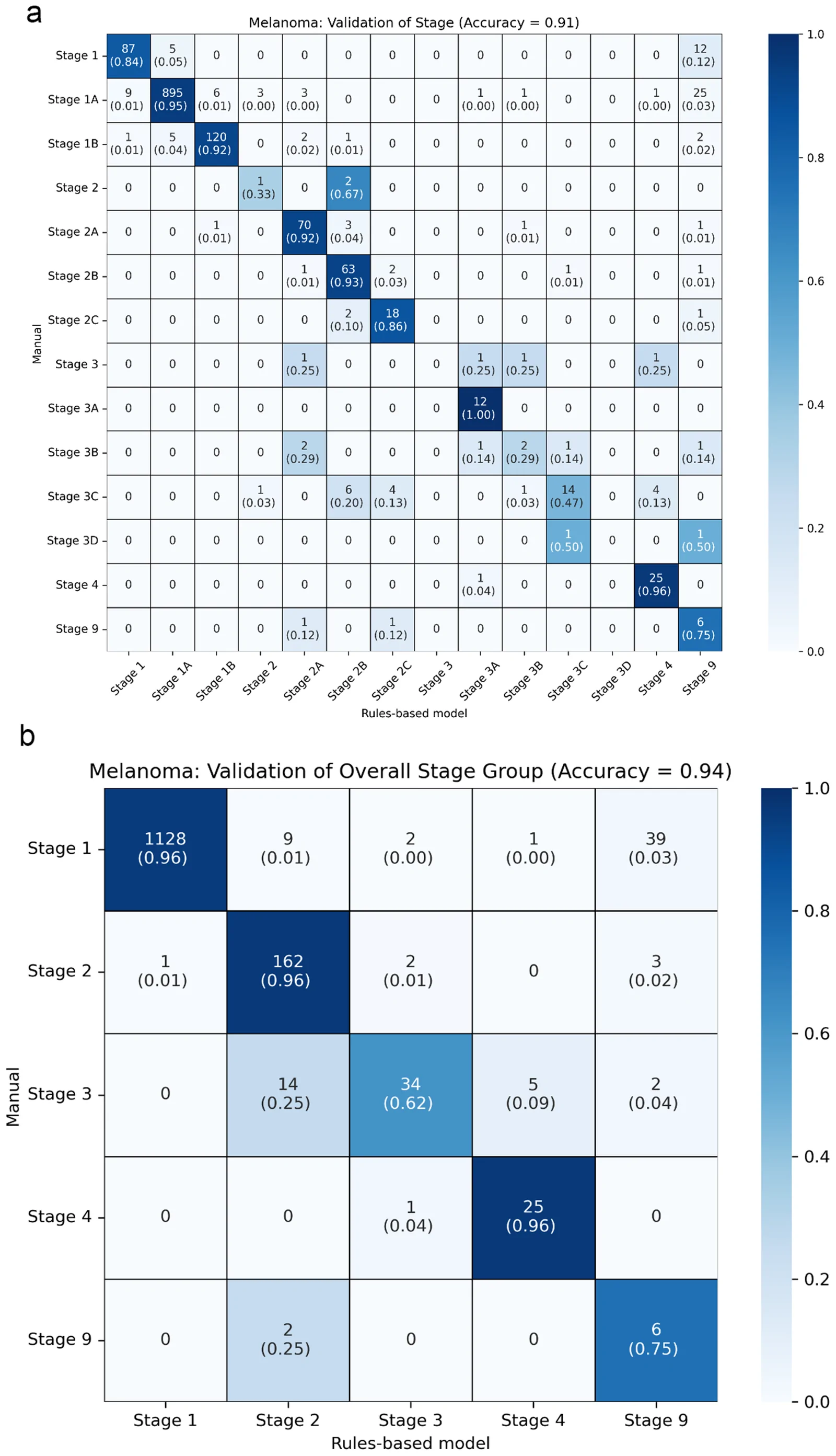
**a**.: Confusion matrix for melanoma AJCC-TNM stage validation based on development corpus . **b**.: Confusion matrix for melanoma overall stage group validation based on development corpus

**Fig. 3:**
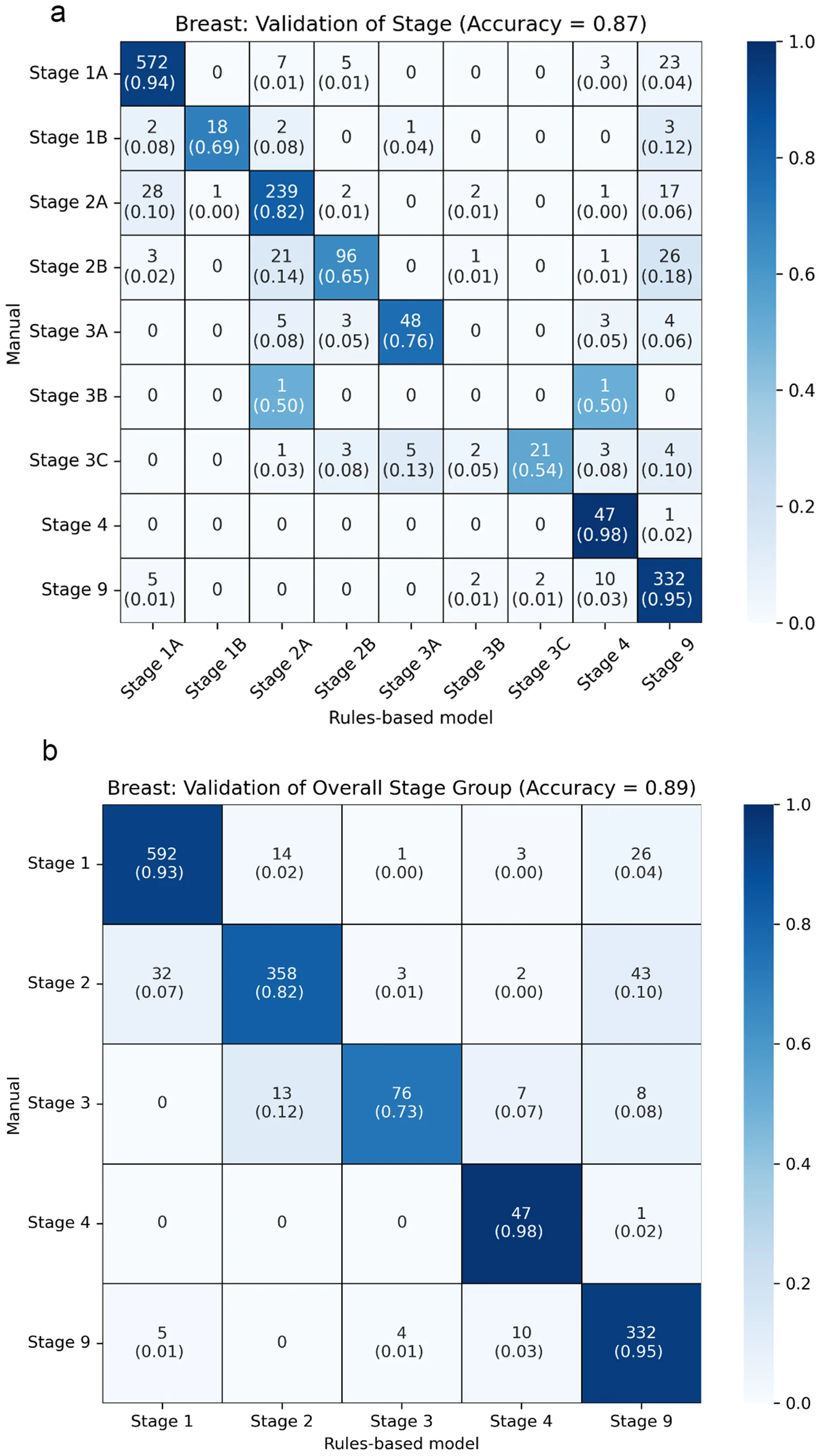
**a**.: Confusion matrix for breast AJCC-TNM stage validation based on development corpus. **b**.: Confusion matrix for breast overall stage group validation based on development corpus

**Fig. 4:**
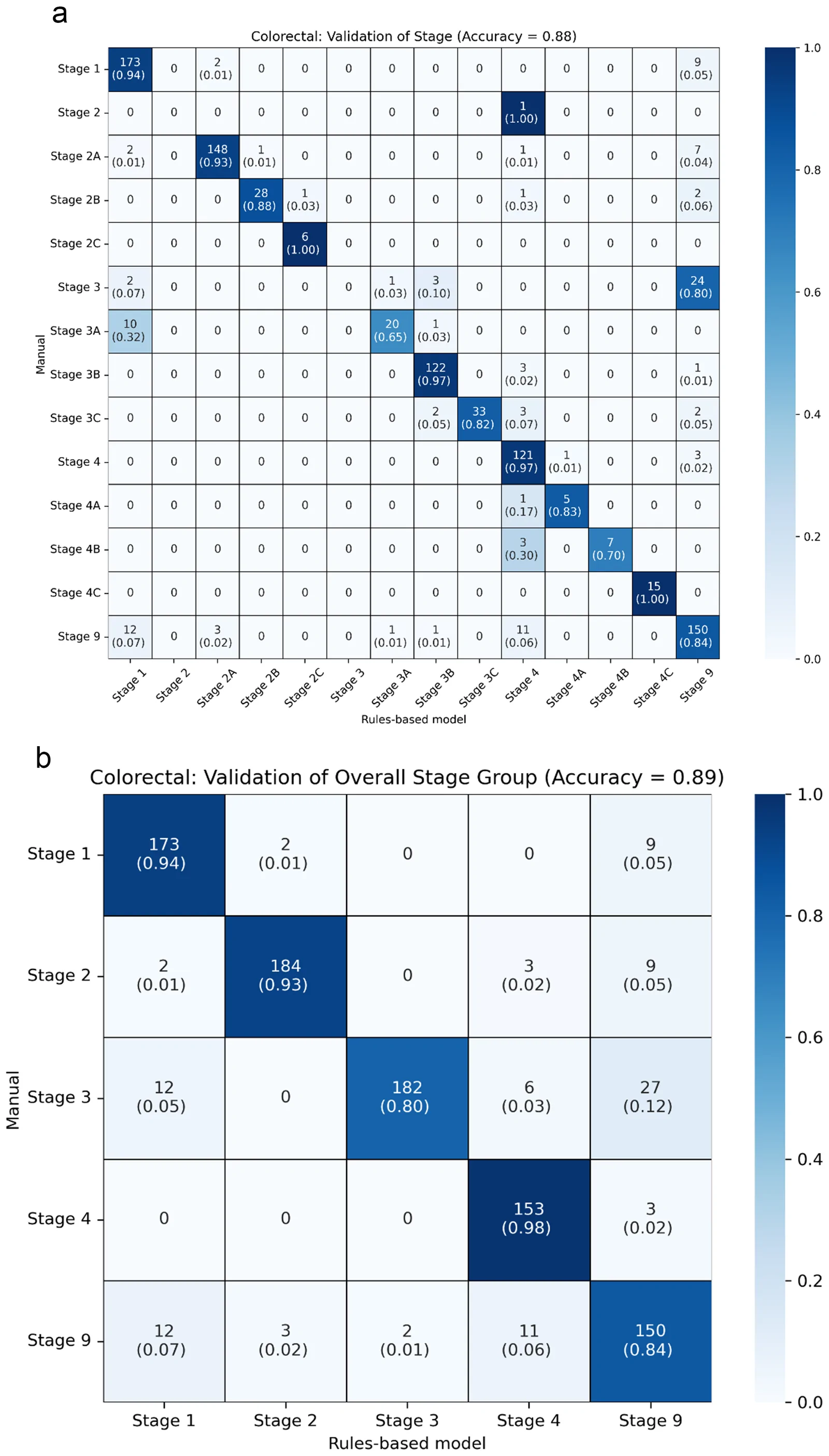
**a**.: Confusion matrix for colorectal AJCC-TNM stage validation based on development corpus. **b**.: Confusion matrix for colorectal overall stage group validation based on development corpus

Detailed breakdowns of the rules-based NLP system accuracy performing AJCC-TNM and overall stage group staging in comparison to the cancer staging project officer are presented in Fig. 2a-b for 2019 melanoma cases, Fig. 3a-b for breast cancer cases, and Fig. 4a-b for colorectal cancer cases. In these matrices, the numbers in each cell of the leading diagonal (top left to bottom right) show the absolute count and proportion where the rules-based NLP system correctly matches the stage, while cells outside this diagonal contain those that are either under-staged or over-staged.

Due to the aggregated nature of overall stage group, the rules-based NLP systems have higher accuracy compared to the more explicit AJCC-TNM (Table 3). As demonstrated in Figs. 2a and 3a, and 4a, AJCC-TNM stages for melanoma, breast and colorectal cancers with low counts were more difficult for the systems to accurately extract information for, compared to more common stages. For example, in Fig. 2a, the melanoma rules-based NLP system’s ability to correctly identify stages 1, 1a, 1b, 2a, 2b, 2c, 3a, and 4 ranged from 84% to 100%; and the total number of these cases in the data ranged from *N* = 12 to *N* = 944. For melanoma AJCC-TNM stages that weren’t accurately staged by the rules-based NLP system (stages 2, 3, 3b, 3c and 3d), the accuracy ranged from 0% to 40%, and the total number of these cases in the data ranged up to *N* = 30.

The melanoma rules-based NLP system had an accuracy of 91% when assigning AJCC-TNM stage, and 94% when assigning overall stage group (Table 3). It understaged 37 (3%) cases when assigning AJCC-TNM stage, and 16 cases (1%) when assigning overall stage group (Fig. 2a and b). Forty cases (3%) were overstaged when assigning AJCC-TNM stage, and 19 cases (1%) when assigning overall stage group. For both the AJCC-TNM stage and overall stage group, the melanoma rules-based NLP system incorrectly staged two unstageable cases (25%); and it incorrectly deemed unstageable 44 cases (88%) that were able to be staged by the cancer staging project officer (Fig. 2a and b).

The breast cancer rules-based NLP system had an accuracy of 87% when assigning AJCC-TNM stage, and 89% when assigning overall stage group (Table 3). When assigning AJCC-TNM stage, 587 cases (37%) were overstaged and 94 cases (6%) were understaged (Fig. 3a and b). Staging performance dramatically improved for assigning overall stage group – 29 cases (2%) were overstaged, and 45 cases (3%) were understaged. For both the AJCC-TNM stage and overall stage group, the breast rules-based NLP system incorrectly staged 19 unstageable cases (5%); and it incorrectly deemed unstageable 78 cases (19%) that were able to be staged by the cancer staging officer.

The colorectal cancer rules-based NLP system had an accuracy of 88% when assigning AJCC-TNM stage, and 89% when assigning overall stage group (Table 3). Eighteen cases (2%) were overstaged when assigning AJCC-TNM stage, and 20 cases (2%) were understaged (Fig. 4a and b). When assigning overall stage group, 11 cases (1%) were overstaged and 14 cases (1%) were understaged. 5% of cases (*N* = 28) that were stageable were incorrectly classified as unstageable by the rules-based NLP system, and 3% of cases (*N* = 48) that were not stageable were incorrectly staged.

The classification reports for overall stage group validation shown in Table 4 further demonstrate the real value of the rules-based NLP system beyond the aggregated accuracy. For stages 1 to 4, the melanoma rules-based NLP system’s precision ranged from 0.80 to 0.99 and recall ranged from 0.62 to 0.96. The breast cancer rules-based NLP system’s precision ranged from 0.68 to 0.94, and the colorectal rules-based NLP system’s precision ranged from 0.87 to 0.99. The recall for melanoma, breast and colorectal cancer were similarly high (ranging from 0,62 to 0.96; 0,73 to 0.98; and 0.80 to 0.98, respectively). The weighted average precision, recall, and F1-scores were 0.97, 0.94, and 0.95 for the melanoma rules-based NLP system; 0,90, 0.89 and 0.89 for the breast cancer rules-based NLP system; and 0.90, 0.89 and 0.89 for the colorectal cancer rules-based NLP system.

**Table 4:** Classification reports for overall stage group validation

**Breast (** ***n*** ** = 1577, accuracy = 0.89)**				
**Stage**	**Frequency**	**Precision**	**Recall**	**F1-score**
Stage 1	636	0.94	0.93	0.94
Stage 2	438	0.93	0.82	0.87
Stage 3	104	0.90	0.73	0.81
Stage 4	48	0.68	0.98	0.80
Stage 9	351	0.81	0.95	0.88
Macro average	1577	0.85	0.88	0.86
Weighted average	1577	0.90	0.89	0.89
**Colorectal (*n* = 947**,** accuracy = 0.89))**				
**Stage**	**Frequency**	**Precision**	**Recall**	**F1-score**
Stage 1	186	0.87	0.94	0.90
Stage 2	198	0.97	0.93	0.95
Stage 3	227	0.99	0.80	0.88
Stage 4	156	0.88	0.98	0.93
Stage 9	180	0.76	0.84	0.80
Macro average	947	0.89	0.90	0.89
Weighted average	947	0.90	0.89	0.89
**Melanoma (*n* = 1436**,** accuracy = 0.94))**				
**Stage**	**Frequency**	**Precision**	**Recall**	**F1-score**
Stage 1	1179	0.99	0.96	0.98
Stage 2	168	0.87	0.96	0.91
Stage 3	55	0.87	0.62	0.72
Stage 4	26	0.80	0.96	0.88
Stage 9	8	0.12	0.75	0.21
Macro average	1436	0.73	0.85	0.74
Weighted average	1436	0.97	0.94	0.95

## Discussion

The rules-based NLP systems developed for overall stages achieved accuracies of 89% for breast cancer, 90% for colorectal cancer, and 94% melanoma. These findings show that the rules-based architecture presented in this study has the potential to improve cancer data collection and staging for population-based cancer registries. Besides the potential to match the level of performance achieved by cancer staging project officers, as demonstrated by the rules-based NLP system accuracy ranging from 89 to 94%, the automated process has a relative advantage of speed thereby ensuring that cancer stage data at diagnosis can be extracted in a timely manner.

Limited synoptic reporting, characterising most of the pathology reports examined in this study, was one of the reasons for discordance between the rules-based NLP system results and the gold standards [6]. Whilst the RCPA have developed general protocols to guide the explicit reporting of tumour features within pathology reports, the way in which these have been implemented differ across laboratories. Non-synoptic reporting results in inconsistent presentation and sometimes omission of essential tumour or nodal characteristics needed to determine the appropriate AJCC-TNM stage via the correct subclasses of T and N values. This also explains why the accuracies were lower for AJCC-TNM stages compared to the overall stage groups for all the cancer streams included in this study. The presence of multiple tumours in a single pathology notification was also noted to decrease/affect the performance of the rules-based NLP system and resulted in under/over staging. Similar observations have been reported by other researchers as well [33]. Without a notable flag to indicate which of the tumours in a single report is the primary tumour, it was difficult to systematically determine in a consistent manner which tumour parameters to extract.

It is acknowledged that the rules-based NLP system developed in this study, despite its promising performance, is resource intensive. The need for rigorous expert rules to guide the entity search underpinning this type of system is a primary limitation for scalability and generalisability. Even where the same entity is being extracted for three cancer types included in this study, the range of values associated with such entity were found to be cancer dependent. For example, melanoma requires 2 or 3 positive nodes to be considered N2, colorectal requires between 4 and 7 while breast requires between 4 and 10 for the same value of N. This downside of rules-based clinical NLP rules-based models is well documented [34]. The commonly recommended solution to this issue is to use advanced models such as deep learning models [35]. One of the strengths of such models that can be appreciated in the development of models with clinical applications are the availability of confidence estimates or uncertainty quantification alongside model predictions, to facilitate downstream quality control by flagging low-confidence cases for review [36]. However, such models are known to be computationally expensive [13], and often inexplainable [37]. The unified system architecture applied in the study is simple and self-explanatory. The rules-based NLP architecture demonstrates a potential for significantly reducing the workload for collecting cancer staging data. Whilst it would be ideal to compare the performance of the rules-based NLP systems to that of a ML model, we lack the labelled data required to generate ML models. This project is potentially part of a larger body of work that may be used to assist the generation of labelled data for training ML models to stage cancer data from pathology reports. Lastly, it is also acknowledged that the rules-based NLP architecture was developed based on cancers diagnosed in Western Australia. Thus, the unavailability of data from other jurisdictions to further test the model’s robustness could be seen as another potential limitation. However, given the performance of our rules-based NLP models on our cross-pathology-provider dataset, it is expectedly robust enough to handle multiple reporting styles.

## Conclusions

The results presented above show that the developed rules-based NLP architecture can be reliably used to undertake TNM staging for melanoma, breast, and colorectal cancers, with or without the T, N, and/or M stages explicitly mentioned. Extensive consultation with knowledge experts was instrumental for maximising the information that could be extracted from pathology reports that contained implicit TNM data. The collection of staging data may be improved by utilising rules-based cancer-specific systems in tandem with those tasked with cancer staging, particularly to improve efficiencies of task and work volume.

## Supplementary Information

Below is the link to the electronic supplementary material.

Supplementary Material 1

Supplementary Material 2

## Data Availability

The datasets generated and analysed during the current study are not publicly available as they are subject to strict data governance that restricts their use to authorised personnel for approved studies. Researchers wishing to access Western Australian Cancer Registry or other health data can apply via the WA Data Linkage System ( [https://www.datalinkageservices.health.wa.gov.au](https://www.datalinkageservices.health.wa.gov.au) ).

## Abbreviations

NLP: Natural language processing
TNM: Tumour, Node, and Metastasis
AJCC: American Joint Committee on Cancer
RCPA: Royal College of Pathologists of Australasia
ML: Machine learning
DNN: Deep neural networks
WA: Western Australia
WACR: Western Australian Cancer Registry
ICD-O: International Classification of Diseases for Oncology
HMDC: Hospital Morbidity Data Collection
ICD-10-AM: International Statistical Classification of Diseases and Related Health Problems, Tenth Revision, Australian Modification
TP: True positive
FP: False positive
FN: False negative
