# Supplementary material for: Cancer staging data collection using rules-based natural language processing for entity extraction from pathology notifications: the WA cancer staging project
Source: BMC Med Inform Decis Mak. 2026 Jun 15;26:322. doi: 10.1186/s12911-026-03617-8 (PMC13495386; doi:10.1186/s12911-026-03617-8)
Supplement: Supplementary file 1 [file 12911_2026_3617_MOESM2_ESM.pdf]

Supplementary Material A.
Business rules developed in consultation with project working groups.

Page 1: ICD-O codes
Page 2: Figure 1. Colorectal Cancer Business Rules.
Page 4: Figure 2. Melanoma Business Rules.
Page 5. Figure 3. Breast Cancer Business Rules.

ICD-O codes

Colorectal cancer
C18.0 C18.5 C18.9
C18.2 C18.6 C19.9
C18.3 C18.7 C20.9
C18.4 C18.8

Breast cancer
C50.0 C50.3 C50.6
C50.1 C50.4 C50.8
C50.2 C50.5 C50.9

Melanoma
C07.9 C44.0 C70.9
C16.2 C44.1 C71.1
C17.0 C44.2 C71.2
C17.2 C44.3 C71.3
C18.0 C44.4 C71.4
C22.0 C44.5 C71.8
C34.0 C44.6 C71.9
C34.1 C44.7 C77.0
C34.2 C44.8 C77.1
C34.3 C44.9 C77.3
C34.9 C49.2 C77.4
C38.2 C49.6 C77.5
C38.3 C64.9 C80.9

Note - refer to figure legends for the included Figures 1-3 for ICD-O codes used for each tumour type.

Regional lymph nodes
C77.0 C77.1 C77.2 C77.3 C77.4 C77.5 C77.8 C77.9

C79.4 C79.6 C79.7 C79.81 C79.82 C79.88 C79.9

Distant metastases
C78.0 C78.1 C78.2 C78.3 C78.4 C78.5 C78.6 C78.7 C78.8 C79.0 C79.1 C79.2 C79.3

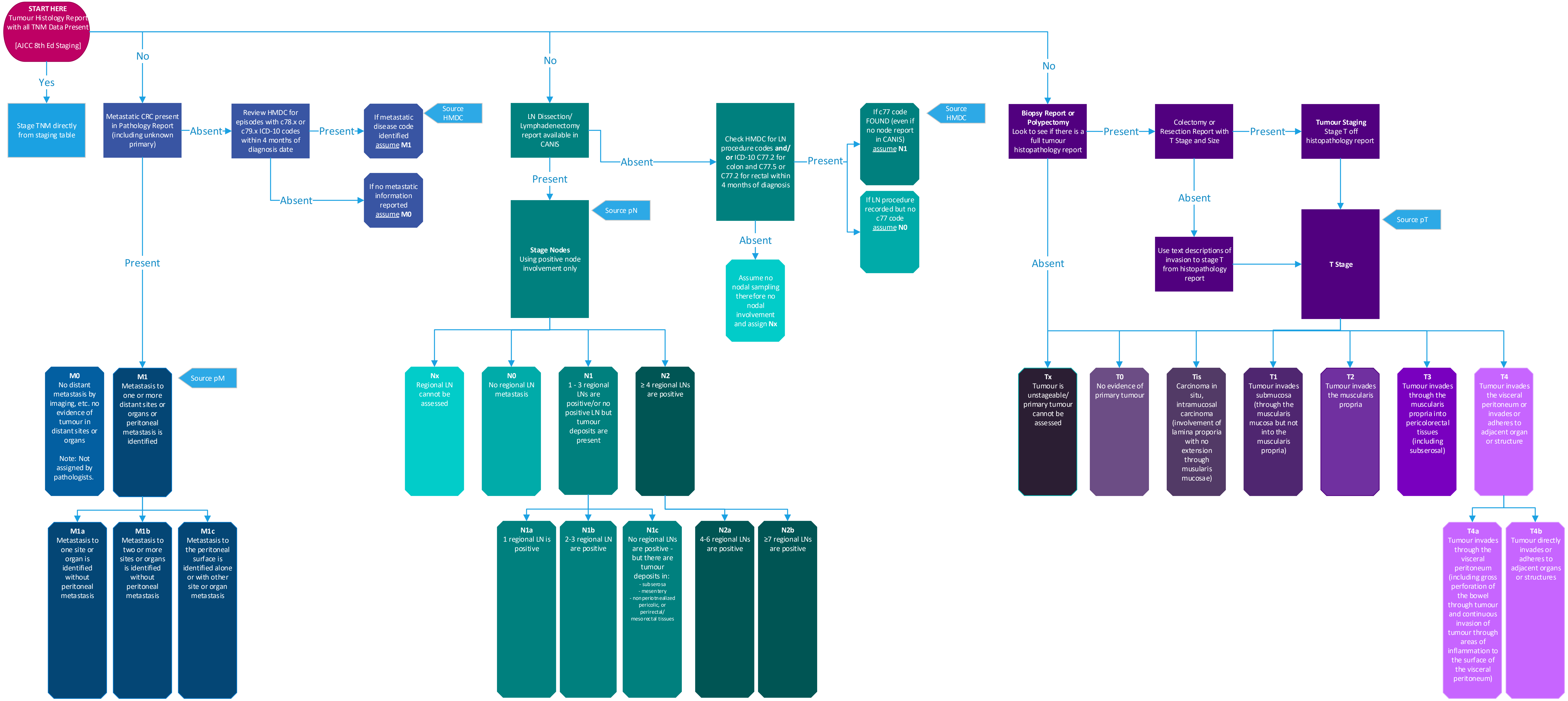

FIGURE 1. COLORECTAL CANCER BUSINESS RULES FOR STAGING COLORECTAL CANCER USING AJCC 8TH EDITION STAGING RULES IN WESTERN AUSTRALIA.

FIGURE 1 LEGEND:
The decision tree outlines the process for assigning AJCC 8th Edition TNM stages for colorectal cancer (CRC) using data from multiple sources.

Abbreviations:

TNM	Tumour, Node, Metastasis staging system
AJCC 8th Ed	American Joint Committee on Cancer, 8th Edition
CRC	Colorectal Cancer
HMDC	Hospital Morbidity Data Collection
CANIS	Cancer Information System (local registry; confirm full name)
pM	Pathologic metastasis classification
pN	Pathologic lymph node classification
pT	Pathologic tumour classification
LN	Lymph Node(s)

ICD-10 – International Classification of Diseases, 10th Revision
C18 – Malignant neoplasm of colon
C19 – Malignant neoplasm of rectosigmoid junction
C20 – Malignant neoplasm of rectum
C77 – Secondary and unspecified malignant neoplasm of lymph nodes
C79 – Secondary malignant neoplasm of other and unspecified types. C79.x indicates all sub-classifications are included (C79.0 through to C79.9)

Staging Categories:

Mx – Metastasis information missing
M0 – No distant metastasis
M1 – Distant metastasis present
- M1a – One site/organ without peritoneal metastasis
- M1b – Two or more sites/organs without peritoneal metastasis
- M1c – Peritoneal surface metastasis with/without other organ metastases

Nx – Nodal involvement information missing
N0 – No regional lymph node metastasis
N1 – 1–3 regional lymph nodes positive
- N1a – 1 node positive
- N1b – 2–3 nodes positive
- N1c – No positive lymph nodes but tumour deposits present

N2 – ≥4 regional lymph nodes positive
- N2a – 4–6 nodes positive
- N2b – ≥7 nodes positive

Tx – Tumour cannot be assessed (Tumour information missing)
T0 – No evidence of primary tumour
Tis – Carcinoma in situ
T1–T4 – Increasing invasion depth
- T4a – Invades through visceral peritoneum
- T4b – Invades/adheres to adjacent organs or structures

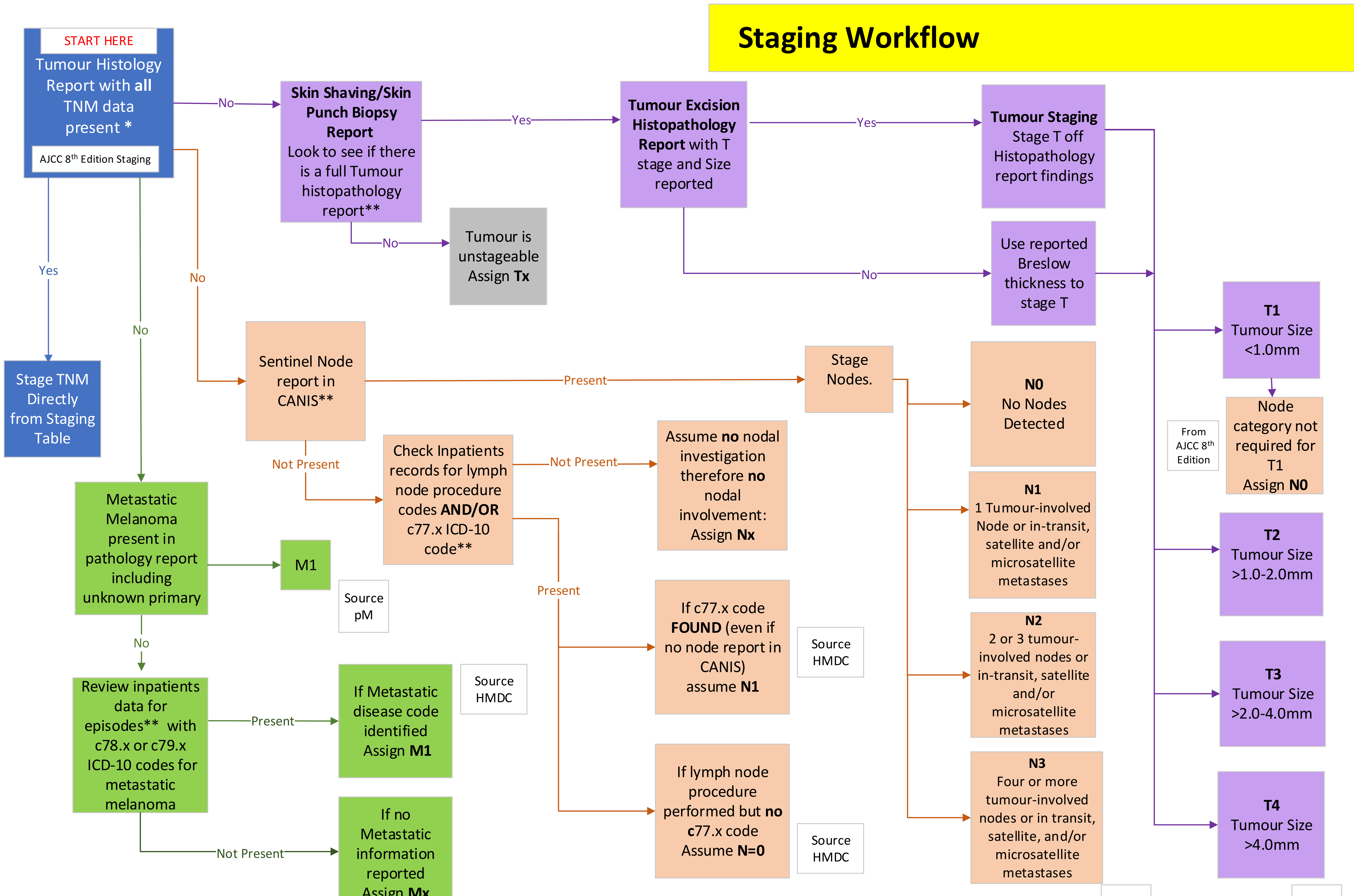

* If more >1 Tumour diagnosis on same day- then stage The largest T
** Within 4 months of melanoma diagnosis

HMDC= Hospital Morbidity Data Collection

Overall Staging

T	N	M	Overall Stage
T1, T1a, T1b	N0	M0	Stage1
T2, T2a			
T2b	N0	M0	Stage 2
T3, T3a, T3b			
T4, T4a, T4b			
Any T	N1, 2, 3	M0	Stage 3
Any T	Any N	M1	Stage 4
Tis, T0, Tx	N0, Nx	M0, Mx	Stage 9*
* Coding Classification when RD Stage cannot be derived			

Assumptions For Overall Stage with X	
When T=X	Assume T0
When N=X	Assume N0
When M=X	Assume M0

FIGURE 2. MELANOMA BUSINESS RULES FOR STAGING MELANOMA USING AJCC 8TH EDITION STAGING RULES IN WESTERN AUSTRALIA.

FIGURE 2 LEGEND:
The decision tree outlines the process for assigning AJCC 8th Edition TNM stages for melanoma using data from multiple sources.

Abbreviations:

TNM	Tumour, Node, Metastasis staging system
AJCC 8th Ed	American Joint Committee on Cancer, 8th Edition
HMDC	Hospital Morbidity Data Collection
CANIS	Cancer Information System (local registry; confirm full name)
pM	Pathologic metastasis classification
pN	Pathologic lymph node classification
pT	Pathologic tumour classification

ICD-10 – International Classification of Diseases, 10th Revision
C77.X – Secondary and unspecified malignant neoplasm of lymph nodes. C77.X indicates all sub-classifications are included (C77.0 though to C77.9)
C78.X – Secondary malignant neoplasm of respiratory and digestive organs. C78.X indicates all sub-classifications are included (C79.0 through to C79.8)
C79.X – Secondary malignant neoplasm of other and unspecified types. C79.X indicates all sub-classifications are included (C79.0 through to C79.9)

Staging Categories:

Mx – Metastasis information missing
M0 – No distant metastasis
M1 – Distant metastasis present
- M1a – One site/organ without peritoneal metastasis
- M1b – Two or more sites/organs without peritoneal metastasis
- M1c – Peritoneal surface metastasis with/without other organ metastases

Nx – Nodal involvement information missing
N0 – No regional lymph node metastasis
N1-N3 – increasing nodal involvement
Tx – Tumour cannot be assessed (Tumour information missing)
T0 – No evidence of primary tumour
T1–T4 – Increasing tumour size

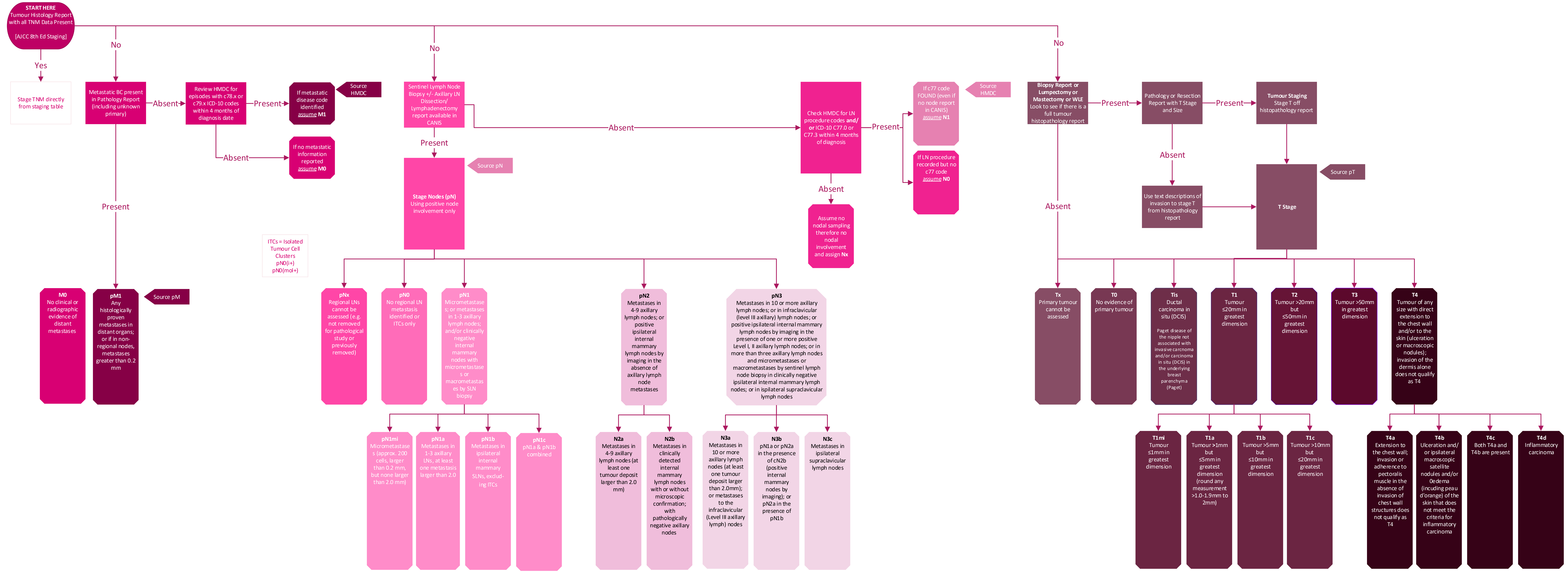

FIGURE 3. BREAST CANCER BUSINESS RULES FOR STAGING BREAST CANCER USING AJCC 8TH EDITION STAGING RULES IN WESTERN AUSTRALIA.

FIGURE 3 LEGEND:
The decision tree outlines the process for assigning AJCC 8th Edition TNM stages for breast cancer using data from multiple sources.

Abbreviations:

TNM	Tumour, Node, Metastasis staging system
AJCC 8th Ed	American Joint Committee on Cancer, 8th Edition
BC	Breast Cancer
HMDC	Hospital Morbidity Data Collection
CANIS	Cancer Information System (local registry; confirm full name)
pM	Pathologic metastasis classification
pN	Pathologic lymph node classification
pT	Pathologic tumour classification
ITCs	Isolated Tumour Cell Clusters
pN0 (i+)	ITCs identified in lymph node
pN0(mol+)	no regional lymph node metastases were found by histology or immunohistochemistry (pN0), but positive molecular findings (e.g., RT-PCR) were detected.
SLN	Sentinel Lymph Node
LN	Lymph Node
WLE	Wide Local Excision
DCIS	Ductal Carcinoma In Situ

ICD-10 – International Classification of Diseases, 10th Revision
C77.X – Secondary and unspecified malignant neoplasm of lymph nodes. C77.X indicates all sub-classifications are included (C77.0 though to C77.9)
C78.X – Secondary malignant neoplasm of respiratory and digestive organs. C78.X indicates all sub-classifications are included (C79.0 through to C79.8)
C79.X – Secondary malignant neoplasm of other and unspecified types. C79.X indicates all sub-classifications are included (C79.0 through to C79.9)

Staging Categories:

M0 – No distant metastasis
M1 – Distant metastasis present
- M1a – One site/organ without peritoneal metastasis
- M1b – Two or more sites/organs without peritoneal metastasis
- M1c – Peritoneal surface metastasis with/without other organ metastases

Mx – Metastasis information missing
Nx – Nodal involvement information missing
N0 – No regional lymph node metastasis
N1-N3 – increasing nodal involvement
Tx – Tumour cannot be assessed (Tumour information missing)
T0 – No evidence of primary tumour
T1–T4 – Increasing tumour size
