# Supplementary material for: Cancer staging data collection using rules-based natural language processing for entity extraction from pathology notifications: the WA cancer staging project
Source: BMC Med Inform Decis Mak. 2026 Jun 15;26:322. doi: 10.1186/s12911-026-03617-8 (PMC13495386; doi:10.1186/s12911-026-03617-8)
Supplement: Supplementary file 2 [file 12911_2026_3617_MOESM1_ESM.docx]

**Supplementary Material B – Pseudocode**

**BEGIN**

## Step 0: Select Tumour Stream ##

Prompt user to select tumour stream:

    	Options: Melanoma, Colorectal, Breast

Set tumour_stream = user selection

## Step 1: Data Extraction & Pre-processing ##

Extract pathology report

Apply text normalization

Apply regular expression (RegEx) patterns

**IF** TNM is explicitly stated **THEN**

Assign overall stage group

**ELSE**

## Step 2: Rules-Based Model ##

**IF** pT is NOT explicitly stated **THEN**

Get tumour information

Assign pT-value using AJCC 8th edition

Proceed to TNM staging

**ELSE**

Return pT value

**IF** pN is NOT explicitly stated **THEN**

Get number of involved lymph nodes and diagnosis

**IF** pN-value is NOT assigned **THEN**

Use ICD-10 codes from HMDC to make N-value assumptions

**ELSE**

Return pN value

Proceed to TNM staging

**IF** pM is NOT explicitly stated **THEN**

Get textual description of distant metastases

**IF** pM-value is NOT assigned **THEN**

Use ICD-10 codes from HMDC to make M-value assumptions

**ELSE**

Return pT value

Proceed to TNM staging

## Step 3: Validation ##

Manual review and update RegEx patterns

**IF** automated stage matches manual stage **THEN**

    Add case to ground truth dataset

**ELSE**

    Perform human review

    Update RegEx rules as needed

**END**

**SUPPLEMENTARY MATERIAL B LEGEND:** Pseudocode outlining the automated algorithm for determining cancer stage from pathology reports using a rules-based natural language processing approach. The algorithm begins with tumour stream selection (melanoma, colorectal, or breast cancer) and applies text normalisation and regular expression patterns to extract TNM staging components. When TNM staging is not explicitly stated, the model systematically determines pT (primary tumour), pN (regional lymph nodes), and pM (distant metastases) values using AJCC 8th edition criteria and ICD-10 code assumptions from Hospital Morbidity Data Collection (HMDC) records. The final validation step compares automated staging results against manual review to iteratively improve pattern recognition accuracy and build a ground truth dataset for model refinement.
